# Metabolites profiling of *Mimusops caffra* leaf via multiplex GC-MS and UPLC-MS/MS approaches in relation to its antioxidant and anti-inflammatory activities

**DOI:** 10.1038/s41598-025-97161-6

**Published:** 2025-04-29

**Authors:** Mostafa H. Baky, Sara M. Rashad, Omayma Elgendy, Safwat A. Ahmed

**Affiliations:** 1 Department of Pharmacognosy, Faculty of Pharmacy, Egyptian Russian University, Badr, Cairo 11829 Egypt; 2 Department of Pharmacognosy, Faculty of Pharmacy, Suez Canal University, Ismailia, Egypt

**Keywords:** *Mimusops caffra*, Sapotaceae, GC-MS, UHPLC-MS, Antioxidant activity, Anti-inflammatory, Drug discovery and development, Secondary metabolism

## Abstract

*Mimusops caffra* is a small to medium-sized fruit-producing tree belonging to the Sapotaceae family with potential commercial, medicinal, and nutritional value. The main goal of the current study is to profile the phytochemical composition of *M. caffra* leaf targeting both volatile and non-volatile metabolites using gas chromatography and mass spectrometry (GC-MS) and ultrahigh performance liquid chromatograpy coupled with mass spectrometry (UPLC-MS/MS), respectively. A total of 62 secondary metabolites were annotated via UPLC-MS/MS belonging to organic acids, phenolic acids, flavonoids, triterpenes, fatty acids and their derivatives, and sphingolipids. Moreover, 50 volatile compounds were detected by using GC-MS classified as monoterpene, aliphatic and aromatic hydrocarbons, alcohols, phenols, fatty acids/esters, and triterpenes. The antioxidant and anti-inflammatory activities of the crude methanol extract, ethyl acetate, *n*-butanol fractions were evaluated using DPPH radical scavenging capacity and nitric oxide inhibition activity, respectively. The crude methanol extract exhibited the strongest antioxidant activity as compared to ethyl acetate, *n*-butanol fractions and ascorbic acid (used as a reference antioxidant). The IC_50_ values of the crude methanol extract, ethyl acetate, *n*-butanol fractions in DPPH assay were 9 ± 0.37 µg/ml, 22.1 ± 0.79 µg/ml and 42.2 ± 1.65 µg/ml, respectively, compared to 12.5 ± 0.7 µg/ml for ascorbic acid. Furthermore, NO inhibition assay revealed that most of tested extracts exhibited marked inhibition (78–88%) at a dose of 1280 µg/mL, the crude methanol extract showed the most potent anti-inflammatory activity with IC_50_ of 137 µg/ml. Overall, these findings suggest that the crude methanol extract, *n*-butanol and ethyl acetate fractions of *M. caffra* contain potential antioxidant compounds highlighting their therapeutic potential.

**Supplementary Information:**

The online version contains supplementary material available at 10.1038/s41598-025-97161-6.

## Introduction

Recently, metabolite profiling of medicinal plants is increasingly applied to assess the bioactive metabolites contributes to their nutritional and health value^1^. Sapotaceae is a family of flowering plants comprising about 1250 species of evergreen trees and shrubs distributed in 53 genera^2^. Several classes of phytochemicals including saponins, flavonoids and polyphenolic compounds, have been reported in Sapotaceae^3^. Moreover, different Sapotaceae species exhibit several biological activities such as antioxidant, anti-inflammatory, antibacterial, antiulcer, antidiabetic, and antifungal^3^. Among Sapotaceae genera, *Mimusops* is a tropical genus consisting of 57 species, many of which produce high-quality timber and edible fruit with significant nutritional and economic value. Owing to their bioactive properties including antioxidant, anti-inflammatory and antibacterial properties^4^, several species have been used to treat various ailments^5^.

*Mimusops caffra* E. Mey. ex A.DC, commonly known as Coastal Red Milkwood is a small to medium-sized fruit-bearing tree distributed in in KwaZulu-Natal, South Africa and the Former Transkei Region, Southern Africa^6^. Additionally, this tree forms up to 75% of the Coastal and Dune Forest in Mozambique^7^. *M. caffra* is also cultivated in Egypt at the Agricultural Research Center garden in Giza governorate, Egypt. The plant is well known for its commercial, ecological, and nutritional value^6^. The fruits of *M. caffra* are fleshy, bright orange-red when mature, edible and pleasantly sweet. The fruit pulp is rich in sucrose, glucose, and fructose with relatively low protein (5.65%) and lipid (6.76%) content. In the food industry, *M. caffra* fruit pulp is used for jelly and alcohol production. Traditionally, *M. caffra* extracts and decoctions have been widely used in ethnomedicine. In Zululand, South Africa, its bark extract is applied to treat wounds and sores, while bark maceration is used as an emetic. The root extracts are also used in the treatment of sexually transmitted infections such as gonorrhea. Additionally, *M. caffra* leaf extract has demonstrated anti-plasmodial and used to manage malaria^8^.

Recently, metabolomics tools have been widely applied for profiling of plant secondary metabolites^9^. The profiling of volatile metabolites in different plants of nutritional and economical value has been extensively reported to assess their quality^10^. Gas chromatography-mass spectrometry (GC-MS) is adopted for analysis of volatile compounds in different plant parts^11,12^. Volatile compounds are typically extracted by using either distillation and/or solvent extraction and most of them have been used widely for different biological activities^13^. *n*-Hexane is highly non-polar and volatile making it suited for extraction of non-polar volatile compounds while minimizing matrix interference in GC-MS analysis. Recently, dimethyl carbonate (DMC) solvent as an alternative to *n*-hexane for extracting volatiles due to its low eco-toxicity compared with *n*-hexane-extracted oil^14^. Unlike different GC methods, hyphenated technique such as ultra-high performance liquid chromatography (UHPLC) with Mass spectrometry is well adopted for profiling of non-volatile polar secondary metabolites^1^.

Despite the significant economic and nutritional values of *M. caffra*, studies on its phytochemical composition remain limited. Therefore, the main goal of this study is to profile the volatile and non-volatile secondary metabolites in *M. caffra* leaves by using GC-MS and LC-MS/MS analysis, respectively. To the best of our knowledge, this work presents the first comprehensive phytochemical profiling of *M. caffra* leaves. Additionally, the antioxidant and anti-inflammatory activities of *M. caffra* leaf methanol extract, *n*-butanol, and ethyl acetate extracts were evaluated using DPPH radical scavenging and NO inhibitory assays.

## Results and discussion

### Metabolites profiling of volatiles in *M. caffra* leaf via GC-MS analysis

**Fig. 1:**
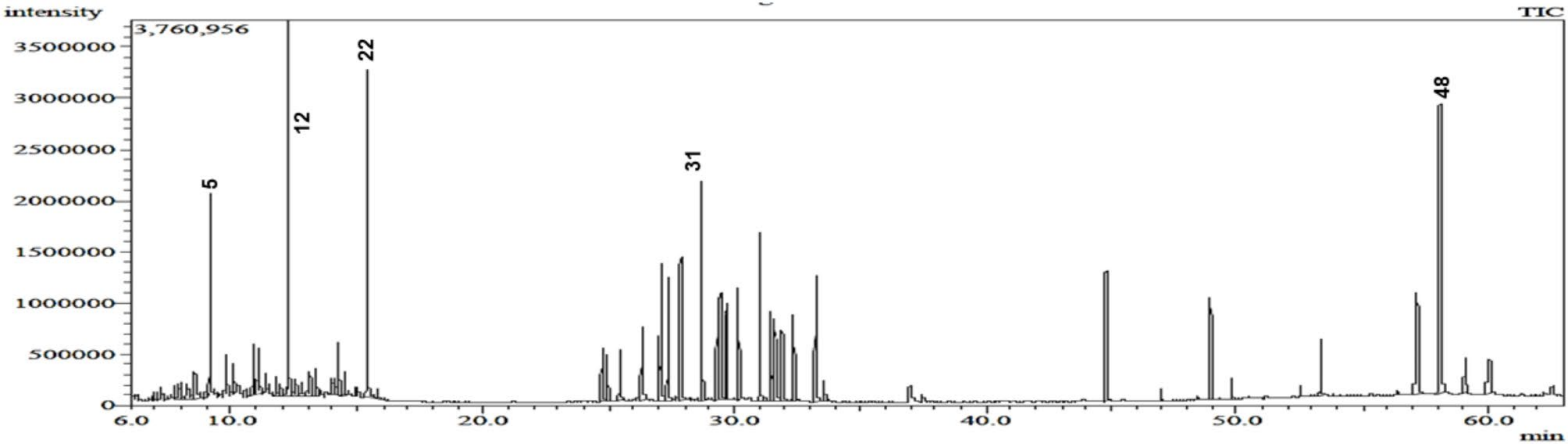
GC-MS Chromatogram of *M. caffra* leaves *n-*hexane extract, **5**: Decane **12**: undecane, **22**: Dodecane, **31**: 2-phenyl undecane and 48: 24-Norursa-3,12-diene.

**Fig. 2:**
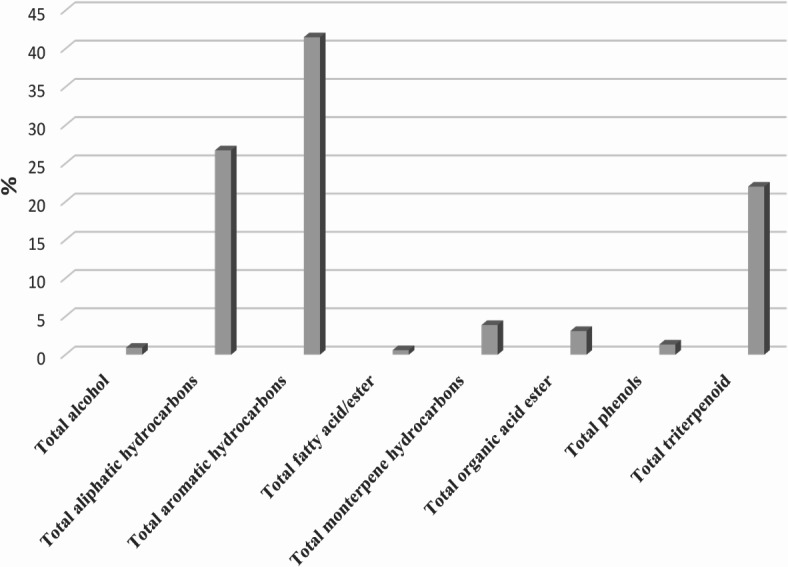
Chemical composition of *M. caffra* leaves hexane extracts.

**Fig. 3:**
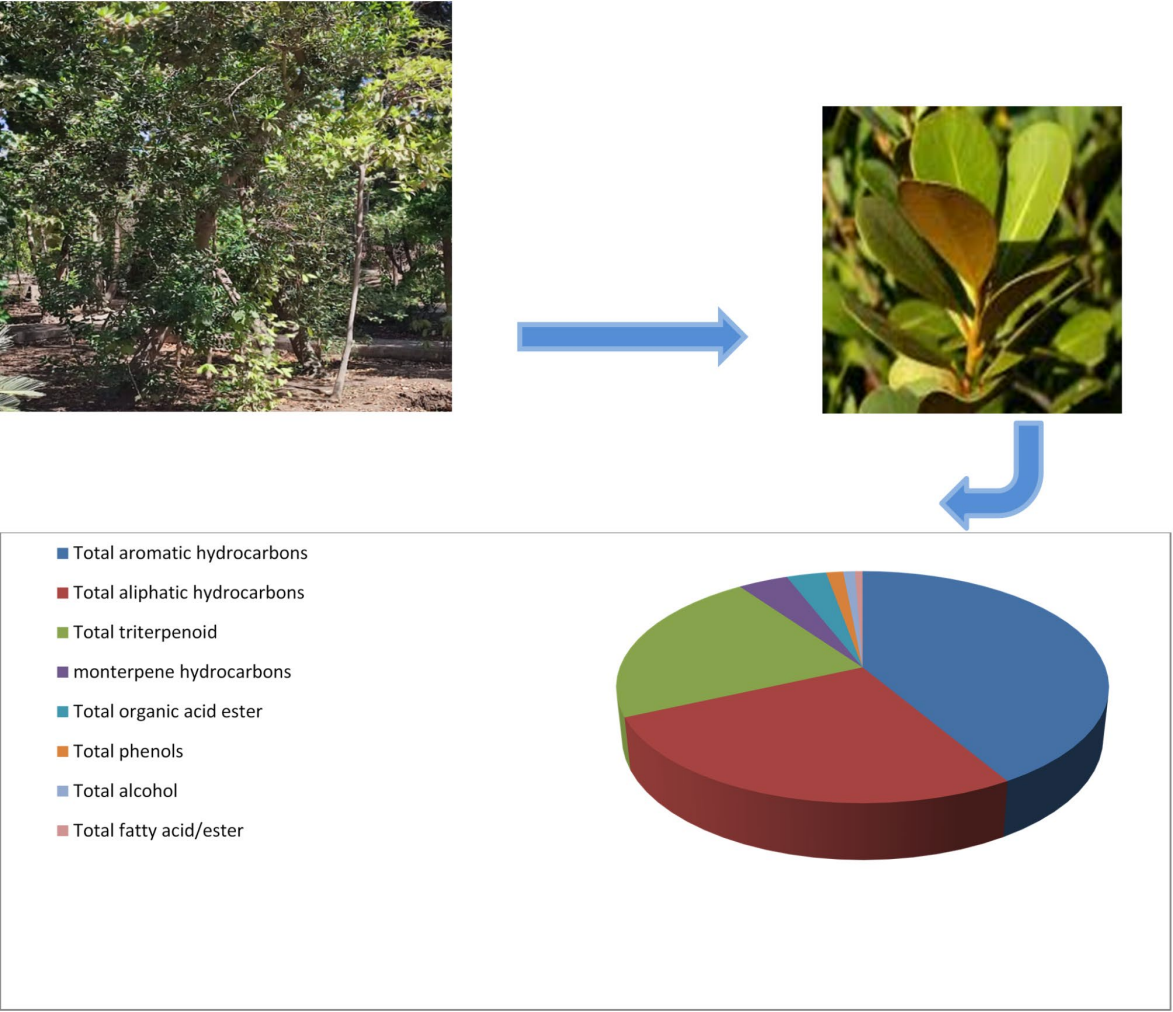
*Mimusops caffra* tree and its leaves and pie charts of different volatile metabolites classes.

The GC-MS analysis of *M. caffra n*-hexane extract led to the identification of 50 compounds (Fig. 1) belonging to various classes including monoterpene, aliphatic and aromatic hydrocarbons, alcohols, phenols, fatty acids/esters, and triterpenes (Table 1; Figs. 2 and 3). The GC-MS chromatogram, displaying the identified compounds and their corresponding peaks, is shown in Fig. 1. The phytoconstituents along with their retention time (RT) and concentration (peak area percentage), are presented in Table 1.

**Table 1:** Relative percentile of volatile metabolites detected in *Mimusops caffra* leaves hexane extracts analyzed using GC–MS.

Peak no	RT	Compound	Class	%	MS
Total monoterpenes hydrocarbons					3.87
1	7.872	1-Ethyl-2,4-dimethylcyclohexane	Monoterpene	0.63	69
2	8.245	1-*p*-Menthene	Monoterpene	1.41	55
11	11.95	1-Ethyl-2-propyl-cyclohexane	Monoterpene	0.53	69
13	12.595	2-Methyldecalin	Monoterpene	0.69	81
16	13.358	Pentyl cyclohexane	Monoterpene	0.61	83
Total aliphatic hydrocarbons					26.7
3	8.567	1-Methyl-3-propylcyclohexane	Aliphatic hydrocarbons	1.09	97
4	8.981	1-Methyl-2-propylcyclohexane	Aliphatic hydrocarbons	0.21	97
5	9.122	Decane	Aliphatic hydrocarbons	3.4	43
6	10.108	Butylcyclohexane	Aliphatic hydrocarbons	0.63	83
7	10.824	Decalin	Aliphatic hydrocarbons	1.09	67
8	11.146	2-Methyldecane	Aliphatic hydrocarbons	1.37	43
9	11.353	3-Methyl decane	Aliphatic hydrocarbons	0.31	57
10	11.768	1-Ethyl-1-methyl-cyclohexane	Aliphatic hydrocarbons	0.54	97
12	12.294	Undecane	Aliphatic hydrocarbons	7.61	57
14	12.814	Dodecane	Aliphatic hydrocarbons	0.73	57
17	14.022	3,6-Dimethyldecane	Aliphatic hydrocarbons	0.61	57
18	14.14	4-Methyl-undecane	Aliphatic hydrocarbons	0.35	43
19	14.274	2-Methyl-undecane	Aliphatic hydrocarbons	1.23	43
20	14.483	3-Methyl-undecane	Aliphatic hydrocarbons	0.56	57
22	15.394	Dodecane	Aliphatic hydrocarbons	6.31	57
23	15.805	3,6-Dimethyl-undecane	Aliphatic hydrocarbons	0.25	57
45	49.847	Tetratetracontane	Aliphatic hydrocarbons	0.42	57
Total aromatic hydrocarbon					41.5
24	24.745	5-Phenyl decane	Aromatic hydrocarbons	1.09	91
25	24.961	4-Phenyldecane	Aromatic hydrocarbons	1.02	91
26	25.42	3-Phenyl decane	Aromatic hydrocarbons	1.25	91
27	26.318	2-Phenyl-decane	Aromatic hydrocarbons	1.81	105
28	27.134	5-Phenyl undecane	Aromatic hydrocarbons	4.12	91
29	27.373	4-Phenyl undecane	Aromatic hydrocarbons	2.51	91
30	27.862	3-Phenyl undecane	Aromatic hydrocarbons	3.08	91
31	28.725	2-Phenyl undecane	Aromatic hydrocarbons	4.35	105
32	29.312	6-Phenyl dodecane	Aromatic hydrocarbons	1.95	91
33	29.422	5-Phenyl dodecane	Aromatic hydrocarbons	2.21	91
34	29.694	4-Phenyl dodecane	Aromatic hydrocarbons	1.9	91
35	30.179	3-Phenyl dodecane	Aromatic hydrocarbons	2.36	91
36	31.031	2-Phenyl dodecane	Aromatic hydrocarbons	3.47	105
37	31.481	6-Phenyl tridecane	Aromatic hydrocarbons	2.35	91
38	31.63	5-Phenyl tridecane	Aromatic hydrocarbons	1.83	91
39	31.902	4-Phenyl tridecane	Aromatic hydrocarbons	1.58	91
40	32.401	3-Phenyl tridecane	Aromatic hydrocarbons	1.84	91
41	33.225	2-Phenyl tridecane	Aromatic hydrocarbons	2.78	105
Total alcohol					0.91
15	13.121	Phytol	Diterpenes alcohol	0.91	43
Total fatty acid/ester					0.59
42	33.54	Palmitic acid, methyl ester	fatty acid/ester	0.59	74
Total organic acid/ester					3.09
21	14.952	Oxalic acid, cyclohexylmethyl isohexyl ester	Organic acid/ester	0.29	97
43	44.81	Phthalic acid, bis(2-ethylhexyl) ester	Organic acid/ester	2.8	149
Total phenols					1.32
46	53.319	α-Tocopherol	Methylated phenols	1.32	165
Total triterpenoid					21.97
44	48.962	Squalene	Triterpenoid	2.03	69
47	57.168	β-Amyrin	Triterpenoid	4.24	218
48	58.104	24-Norursa-3,12-diene	Nortriterpenes	12.31	218
49	59.078	β-Amyrin (12-oleanenol) acetate	Triterpenoid	1.53	218.2
50	60.051	α-Amyrin	Triterpenoid	1.86	218

#### Aromatic hydrocarbons

Aromatic hydrocarbons were the predominant constituents detected in *M. caffra n*-hexane extracts, accounting for 41.5%, of the total composition. The most abundant compounds detected were 2-phenyl undecane (peak 31), 5-phenyl undecane (peak 28), and 2-phenyl dodecane (peak 36) were prevalent by 4.35, 4.12, and 3.47% respectively, Aromatic hydrocarbons and their phenylundecane derivatives have been reported for their notable antifungal and antibacterial activity^15^.

#### Aliphatic and monoterpene hydrocarbons

Aliphatic hydrocarbons constituted the second most abundant volatile class in the *n*-hexane extract of *M. caffra*, accounting for 26.7% of the total identified compounds. The most prevalent compounds were undecane (peak 12), dodecane (peak 22), and decane (peak 5) by 7.61, 6.31, and 3.4%, respectively. Undecane, a naturally occurring alkane hydrocarbon, has been reported to exhibit a potent anti-inflammatory and anti-allergic activities^16^. Dodecane has demonstrated antioxidant properties^17^. Unlike aliphatic hydrocarbons, monoterepene hydrocarbons were detected at lower percentage at 3.87% including 1-*p*-menthene (peak 2), 2-methyldecalin (peak 13), 1-ethyl-2,4-dimethylcyclohexane (peak 1), pentyl cyclohexane (peak 16) and 1-ethyl-2-propyl-cyclohexane (peak 11) were prevalent by 1.41, 0.69, 0.63, 0.61 and 0.53% respectively.

#### Triterpenes and phenols

Triterpenes constitutes 21.97% of the identified compounds in the *n*-hexane extract. Squalene (peak 44), *β*-amyrin (peak 47), 24-norursa-3,12-diene (peak 48), β-amyrin acetate (peak 49) and α-amyrin (peak 50) were the most abundant by 2.03, 4.24, 12.31, 1.53, and 1.86% respectively. Triterpenes *α*-amyrin and *β*-amyrin have been reported to have an antioxidant and a potential anti xanthine oxidase and tyrosinase enzyme inbibtors so used for preventing gout and skin hyperpigmentation respectively^18^ and they were identified in *Manilkara zapota* leaves of Sapotaceae family^19^. Squalene, a hydrophilic natural antioxidant, has demonstrated antiradical and antioxidant properties depending on the model system employed for the study^20^. Its presence in *M. caffra* is consistent with previous reported study on the petroleum ether extract of *Mimusops elengi* leaves, marking the first confirmation of squalene detection in *M. caffra*^21^. Phenols were also detected in the *n*-hexane extracts by α-tocopherol (peak 46) which accounted for 1.32%. α-Tocopherol is the major vitamin E compound found in leaf chloroplasts and has been reported to has a potential antioxidant activity^22^.

#### Organic acid ester

Organic acid esters accounted for 3.09% of the total identified compounds including oxalic acid, cyclohexylmethyl isohexyl ester (peak 21) and phthalic acid, bis(2-ethylhexyl) ester (peak 43) which accounted 0.29 and 2.8% respectively. Phthalic acid esters have been reported to function as allelochemicals, insecticidal, phytotoxic and have a potential antimicrobial activity^23^.

#### Alcohols and fatty acids/esters

Alcohols were detected in trace levels in the *n*-hexane extract of *M. caffra* leaves and represented by phytol (peak 15) 0.91%. Phytol is a diterpene member of the long-chain unsaturated acyclic alcohols, known for its roles as a natural antioxidant. While saturated aliphatic alcohols generally exhibit poor antioxidant activity, phytol exhibits a good antioxidant potential due to the allylic nature of its alcohol group^24^, and it has been identified in different plant species across different families^25^. Likewise, fatty acids/ester represented by palmitic acid, methyl ester (peak 42) 0.59% was detected at trace levels among volatile components of *M. caffra* leaves. It was previously identified in *Manilkara zapota* tree belonging to the Sapotaceae family^26^.

### Secondary metabolites profiling of *M. caffra* leaf via UPLC-MS/MS analysis

**Fig. 4:**
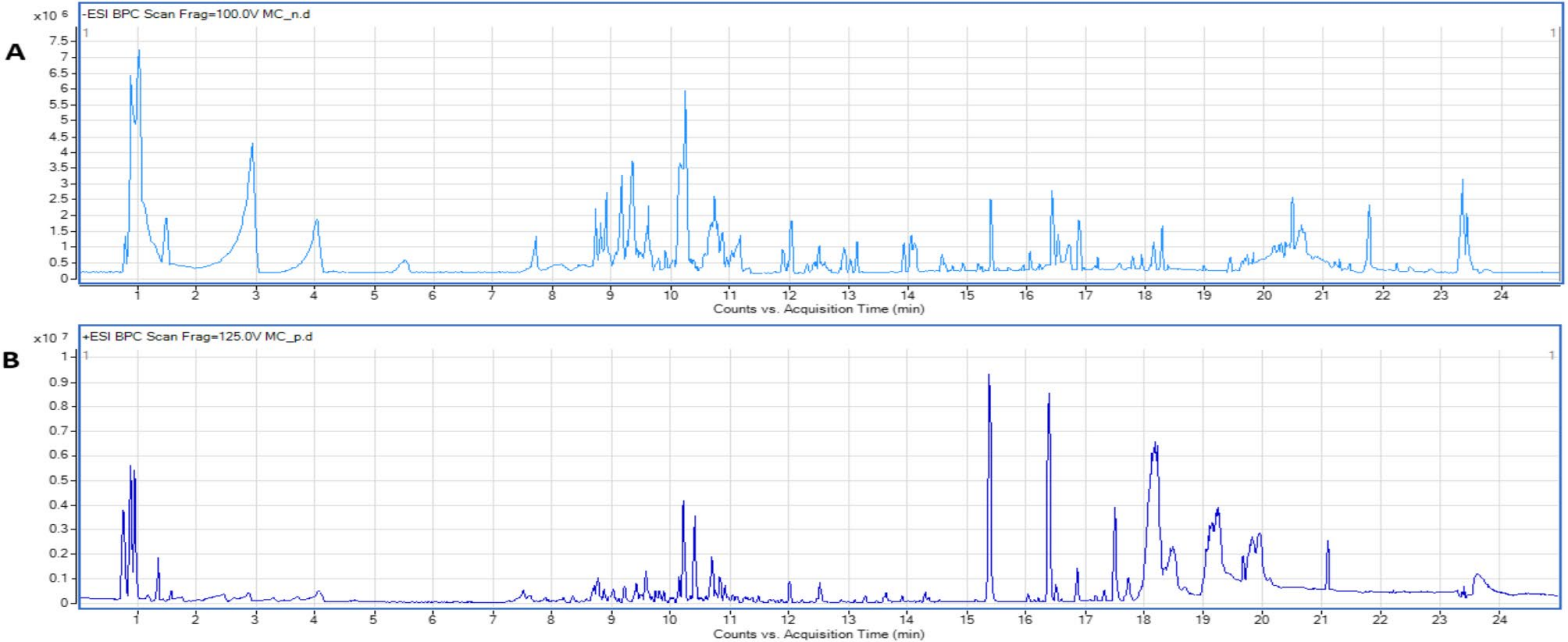
Base peak chromatogram of *M. caffra* leaf extract (**A**) analysis in negative mode (**B**) Positive mode.

UPLC-MS/MS analysis of *M. caffra* leaf crude methanol extract was performed in negative (Fig. 4A) and positive ionization modes (Fig. 4B). UPLC-MS/MS analysis led to the identification of 62 metabolites (Tables 2, 3) belonging to the various phytochemical classes including organic acids, phenolic acids, flavonoids in addition to their derivatives besides the presence of triterpenoids and fatty acids which were reported in negative ionization mode (Table 2). Moreover, phenolic acids, flavonoids were also reported in positive ionization mode beside to trace compounds belong to amino acids, alkaloids and sphingolipids with unknown compounds (Table 3). The order of elution of various chromatographic peaks occurred with decreasing polarity starting with organic acids, and simple phenolics, followed by flavonoid glycosides then aglycones and finally fatty acids and triterpenoids. Phenolic and flavonoid metabolites represented the most abundant class. Inspection of both negative and positive ionization modes revealed a higher detection level in the negative ionization mode especially for phenolic acids, and flavonoids. This is the first detailed metabolites characterization of *M. caffra* using high-resolution UPLC-MS. Therefore, the high content of glycosylated flavonoids and important bioactive phenolic compounds can contribute the value of *M. caffra* leaves as a potent antioxidant and promote beneficial effects on human health and well-being. However, further in vivo studies should be conducted with leaves to validate these effects.

**Table 2:** Chemical metabolites of *M. caffra* characterized by UPLC-Q-Exactive-MS/MS at negative mode.

	Identified/tentatively annotated compound	*R*.t. (min)	Mass (m/z) [M − H]−	Calculated mass	MS/MS fragment ions (m/z)	Molecular formula	Error (ppm)	References
Organic acids								
1	Malic acid	1.026	133.0137	134.0209	115.0022, 89.0234, 71.0139	C_4_H_6_O_5_	4.49	^65^
2	Furoic acid or isomer	1.501	111.0082	112.0155	–	C_5_H_4_O_3_	4.89	^66^
3	Azelaic acid	10.986	187.0968	188.1041	–	C_9_H_16_O_4_	4	^67^
Phenolic acids and its derivatives								
4	Gallic acid	2.344	169.0135	170.0208	125.0139	C_7_H_6_O_5_	4.53	^31^
5	*p*-Hydroxybenzoic acid	10.927	137.0238	138.0311	93	C_7_H_6_O_3_	4.21	^68^
6	Protocatechuic acid	4.631	153.0184	154.0257	109	C_7_H_6_O_4_	5.86	^69^
7	Quinic acid	2.435	191.0551	192.0624	127, 85, 173, 93	C_7_H_12_O_6_	5.38	^35^
8	Galloyl quinic acid isomer I^a^	3.768	343.0657	344.0731	191.0497 169.0125	C_14_H_16_O_10_	3.75	^43^
9	Digalloyl quinic acid isomer IV	8.354	495.0755	496.0827	343.0655, 191.0548 169.0117	C_21_H_20_O_14_	5.26	^43^
10	*p*-Coumaroylquinic acid	8.849	337.0915	338.0987	163.0397, 191.0558	C_16_H_18_O_8_	4.31	^37^
11	Pyrogallol	2.903	125.0238	126.0311	107.0130	C_6_H_6_O_3_	4.95	^70^
12	Dihydroxybenzoic acid hexoside	8.095	315.0723	316.0793	315, 153, 152, 109, 108	C_13_H_16_O_9_	0.54	^42^
13	Vanillic acid hexoside	9.63	329.0895	330.0963	329	C_14_H_18_O_9_	− 3.6	^38^
14	Methyl dihydroxybenzoic acid hexoside	9.63	329.0895	330.0963	329,167, 152,123	C_14_H_18_O_9_		^42^
15	Caffeic acid-*O*-hexoside	10.075	341.0896	342.0965	179, 135	C_15_H_18_O_9_	− 4.07	^39^
16	Syringic acid-*O*-hexoside	9.689	359.0999	360.1066	197, 182, 167, 153, 138, 123, 95	C_15_H_20_O_10_	− 2.77	^39^
17	1,2-Digalloyl-beta-Dglucopyranose	4.308	483.0789	484.0862	289,353,389	C_20_H_20_O_14_	− 1.85	^32^
Flavonoids and its derivatives								
18	Quercetin	11.86	301.0341	302.0413	151, 179	C_15_H_10_O_7_	4.43	^35^
19	Epicatechin	9.552	289.0705	290.0777	245.0794, 203.0661, 151.0388, 109.0300	C_15_H_14_O_6_	4.53	^65^
20	Gallocatechin	8.71	305.0654	306.0726	305, 261, 219, 179, 125	C_15_H_14_O_7_	4.26	^42^
21	Ethyl 2,4 dihydroxy-3-(3,4,5-trihydroxybenzoyl)oxybenzoate	8.564	349.0581	350.065	198.0759,197.0420, 169.0096, 124.0149	C_16_H_14_O_9_	− 3.39	^43^
22	Galloyl hexoside	4.41	331.0682	332.0752	331,169,151, 125	C_13_H_16_O_10_	− 2.66	^42^
23	Ethyl-*O*-β-D-(6ʹ-Ogalloyl)-glucopyranoside	9.689	359.0999	360.1066	169.0140	C_15_H_20_O_10_	− 2.77	^43^
24	Apigenin-7-*O*-B-D-glucoside	11.131	431.0967	432.1039	269.0439	C_21_H_20_O_10_	3.97	^65^
25	Kaempferol-3-*O*-deoxyhexoside	11.131	431.0967	432.1039	431.1002, 285.0408, 284.0332, 255.0303, 227.0352, 229.0506	C_21_H_20_O_10_	3.97	^47^
26	Kaempferol-3-*O*-hexoside	10.697	447.0917	448.0989	447.0945,285.0398, 284.0318, 255.0292, 227.0340, 151.0031	C_21_H_20_O_11_	3.69	^47^
27	Quercetin-3-*O*-hexoside (Isoquercitrin)	10.429	463.0868	464.0939	463.0899, 301.0355, 300.0271, 271.0247, 255.0295, 243.0296, 178.9985, 151.0035	C_21_H_20_O_12_	3.38	^47^
28	Myricetin-3-*O*-hexoside	9.88	479.0808	480.0882	479.0841, 317.0286, 316.0221, 287.0193, 271.0239	C_21_H_20_O_13_	4.61	^47^
Glycosides								
29	Ethyl 7-epi-12-hydroxyjasmonate glucosid	10.667	415.1956	416.2029	389/405	C_20_H_32_O_9_	4.14	^32^
Triterpenes								
30	Pomaceic acid	14.92	501.3196	502.3269	483.5, 457.6, 441.5, 409.7	C_30_H_46_O_6_	5.08	^71^
31	Pomolic acid	16.937	471.3454	472.3528	453.6, 411.6, 407.6	C_30_H_48_O_4_	5.18	
32	Euscaphic acid	14.974	487.3408	488.3481	425.6, 469.5,407.6	C_30_H_48_O_5_	4.24	
Fatty acids								
33	Dodecenedioic acid	12.99	227.1281	228.1353	183.1394; 165.1278	C_12_H_20_O_4_	3.58	^47^
34	Dihydroxyhexadecanoic acid	13.324	287.2214	288.2286	287.2235; 269.2127	C_16_H_32_O_4_	5.22	^47^
35	9-Oxooctadeca-10,12,15-trienoic acid	16.755	291.1949	292.2017	291.1969; 273.1851; 247.2069; 223.1701; 195.1382	C_18_H_28_O_3_	7.41	^47^
36	α-Linolenic acid	18.983	277.2159	278.2232	277.2181	C_18_H_30_O_2_	4.83	^47^
37	13-Oxo-9,11-octadecadienoic acid	17.388	293.2106	294.2179	293.2119, 275.2022, 249.2220, 195.1390, 185.1179, 153.1287, 113.0974	C_18_H_30_O_3_	5.41	^47^
38	13-Hydroxyoctadeca-9,15-dienoic acid	17.876	295.2263	296.2336	295.2284, 277.2176, 183.1389	C_18_H_32_O_3_	5.33	^47^
39	9,12,13-Trihydroxy-10,15- octadecadienoic acid	12.462	327.2165	328.2237	309.2076, 291.1970, 239.1288, 229.1446, 221.1179, 211.1338, 191.1236, 183.1391, 171.1022, 137.0966	C_18_H_32_O_5_	3.76	^47^
40	9-Hydroxyoctadec-12-enoic acid	17.508	297.2423	298.2495	297.2439, 279.2331, 171.1027, 155.1075	C_18_H_34_O_3_	4.5	^47^
41	Dihydroxyoctadecenoic acid	14.879	313.2367	314.2441	313.2377, 295.2270, 277.2163, 183.1384, 129.0916	C_18_H_34_O_4_	5.28	^47^
42	9,12,13-Trihydroxy-10-octadecenoic acid	13.704	329.2315	330.2388	229, 211, 171	C_18_H_34_O_5_	5.58	^47^
Miscellaneous								
43	Glutaconic acid	2.218	129.0188	130.0261	112/119	C_5_H_6_O_4_	3.92	^32^
44	Unknown	7.679	219.0501	220.0573	192.0307, 175.0312, 157.0490, 129.0881, 111.0077	C_8_H_12_O_7_	4.45	^70^
45	N.i	4.672	449.1274	450.1359	271.0503, 169.0157 125.0159	C_18_H_26_O_13_	3.25	^43^
46	Unknown	3.021	339.034	340.0415	169.0138, 125.0235	C_14_H_12_O_10_	4.48	^70^
47	Acetyl-maltose	1.289	383.1179	384.1252	357,365	C_14_H_24_O_12_	4.14	^32^

**Table 3:** Chemical metabolites of *M. caffra* characterized by UPLC-Q-extractive-MS/MS at positive mode.

	Identified compound	Rt (min)	Mass (m/z) [M + H]+	Calculated mass	MS/MS fragment ions (m/z)	Molecular formula	Error (ppm)	References
Phenolic acids								
48	Gallic acid	2.837	171.0287	170.0214	141,151,160	C_7_H_6_O_5_	0.53	^32^
49	Quinic acid	1.023	193.0707	192.0634	174	C_7_H_12_O_6_	0.03	^32^
50	4-(2-Hydroxypropoxy)-3,5-dimethyl-Phenol	10.809	197.1172	196.1099	–	C_11_H_16_O_3_	0.3	^32^
51	4-*p*-Coumaroylquinic acid	8.889	339.1072	338.1	–	C_16_H_18_O_8_	0.6	^32^
Flavonoids								
52	5,7,20,30—Tetrahydroxyflavone	11.127	287.055	286.0477	265,275	C_15_H_10_O_6_	0.18	^32^
53	3,5,7,20,50—Pentahydroxyflavone	10.696	303.05	302.0428	273,289	C_15_H_10_O_7_	− 0.43	^32^
54	Ent-Fisetinidol-4beta-ol	8.991	291.0863	290.079	262	C_15_H_14_O_6_	0.21	^32^
Alkaloids								
55	Gentiatibetine	4.45	166.0861	165.0789	143,151	C_9_H_11_NO_2_	0.74	^32^
Amino acid								
56	l-Tryptophan	8.313	205.0971	204.0899	188.0708, 146.0603, 118.0654	C_11_H_12_N_2_O_2_	0.03	^49^
Sphingolipids								
57	Dehydrophytosphingosine	14.313	316.2843	315.277	298,286, 281, 280, 262, 256, 141	C_18_H_37_NO_3_	0.96	^50^
58	Octadecasphinganine	14.723	302.305	301.2977	285,284, 217	C_18_H_39_NO_2_	1.41	^50^
59	Phytosphingosine	14.761	318.3	317.2923	300, 282, 264	C_18_H_39_NO_3_	2.08	^50^
Miscellaneous								
60	Pyroglutamic acid	1.58	130.0497	129.0421	–	C_5_H_7_NO_3_	3.88	^32^
61	Valine	1.355	118.0862	117.0789	104	C_5_H_11_NO_2_	0.68	^32^
62	2-Amino-3-methyl-1-butanol	0.951	104.1072	103.1	–	C_5_H_13_NO	− 2.46	^32^

*N.i* not identificated, *r.t* retention time.

The identified metabolites are listed in Tables 2 and 3 which represents the negative and positive ionization mode, respresentively. Results revealed a total of 62 metabolites identified in the crude methanol extract, including organic acids (3 metabolites) which are identified in the negative mode only, phenolic acids and their derivatives (14 and 4 metabolites) in the negative mode and positive mode representively, flavonoids and flavonoidal glycosides (11 and 3 metabolites) in the negative mode and positive mode representively, as well as triterpenes (3 metabolites) and fatty acid derivatives (10 metabolites) which are identified in the negative mode only. amino acids, alkaloids and sphingolipids along with unknown compounds were reported in positive ionization mode only.

#### Organic acids

Organic acids and derivatives (compounds 1–3) were tentatively identified in *M*. *caffra* methanol extract, eluting early in the chromatogram among which malic acid (peak 1) (133.0137, C_4_H_6_O_5_), 2-furoic acid (peak 2) (111.0082, C_5_H_4_O_3_), and azelaic acid (peak 3) (187.0968, C_9_H_16_O_4_) were identified in the negative ionization mode and malic acid has already been widely reported in previous studies^27^. Organic acids belong to an important class of organic compounds that contribute to the flavor of fruits and vegetables. malic acid is one of the main organic acids responsible for the flavor notes of most fruits^28^.

#### Phenolic acids and its derivatives

Phenolic compounds represent a diverse class of organic compounds characterized by their aromatic nature and the presence of one or more hydroxyl groups attached to the phenyl ring^29^. These compounds have garnered significant attention due to their various physiological and pharmacological properties. Phenolics are widely distributed throughout the plant kingdom and serve as powerful antioxidants and exhibiting potential benefits for human health^30^. *M. caffra* leaves have been identified to contain a diverse array of phenolic compounds such as gallic acid (C_7_H_6_O_5_) in peaks 4 and 48 at *m/z* (169.0135 and 171.0287) which has been identified according to^31,32^. Gallic acid, one of the hydroxybenzoic acids, was reported in previous studies as a polyphenolic antioxidant in fruits of *Pouteria* species belongs to Sapotaceae family^33^. Among phenolic acids, gallic acid is tremendously well absorbed into the human body, compared with other polyphenols. It was shown to have a positive effect against cancer cells under in vitro conditions^34^. In addition, quinic acid (C_7_H_12_O_6_) in peaks 7 and 49 at *m/z* (191.0551 and 193.0707) has been identified as follows^32,35^. It was previously reported in methanol extract of *Mimusops elengi* with [M-H]^−^ at *m/z* 191.0564^36^, confirming quinic acid detection in *caffra* species which was reported for the first time. Moreover, *p*-coumaroylquinic acid (C_16_H_18_O_8_) in peaks 10 and 51 at *m/z* (337.0915 and 339.1072) has been identified according to^32,37^. The previous phenolic compounds have been identified in the negative and positive ionization modes respectively. Some of the phenolic compounds present in the leaves (compounds 12–17) were found to be linked to one or more sugar residues where they are identified in the negative ionization mode only such as vanillic acid hexoside, caffeic acid-*O*-hexoside, syringic acid-*O*-hexoside in peaks 13, 15, and 16 with [M-H]^−^ at *m/z* (329.0895, C_14_H_18_O_9_-) with MS^2^ fragments at *m/z* 329, ( 341.0896, C_15_H_18_O_9_-) with MS^2^ fragments at *m/z*179 and135 and (359.0999, C_15_H_20_O_10_-) with MS^2^ fragments at *m/z* 197, 182, 167, 153, 138, 123, 95 respectively, vanillic acid-*O*-hexoside has been identified according to^38^. While caffeic acid-*O*-hexoside and syringic acid-*O*-hexoside according to^39^. Vanillic acid-*O*-hexoside and syringic acid-*O*-hexoside have been reported in previous studies to have antioxidant activity^40^.

#### Flavonoids and its derivatives

Flavonoids are a group of natural polyphenolic compounds consist of a flavan nucleus composed of two benzene rings linked by a heterocyclic pyran ring or pyrone, they have a powerful antioxidant and anti-inflammatory activities^41^. The subclasses of flavonoids commonly found in plants include flavones, flavanones, flavonols, and isoflavones, A total of 14 flavonidal compounds identified in both negative and positive mode (11 and 3), respectively. The identified free flavonoids including quercetin, epicatechin, gallocatechin and ethyl 2,4 dihydroxy-3-(3,4,5-trihydroxybenzoyl)oxybenzoate were determined in this study in peaks (18–21) with [M-H]^−^ at *m/z* ( 301.0341, C_15_H_10_ O_7_-), (289.0705, C_15_H_14_O_6_^_^), (305.0654, C_15_H_14_O_7_-) and (349.0581, C_16_H_14_O_9_^_^) and exhibiting MS^2^ fragments at *m/z* 151 and 179^35^, 245.0794, 203.0661, 151.0388, 109.0300^35^, 305, 261, 219, 179, 125^42^ and 198.0759,197.0420, 169.0096, 124.0149^43^ respectively. Epicatechin has shown different pharmacological activities such as antiviral^44^ and antioxidant^45^ activities. Recently, epicatechin has been reported to has a potential use in the management of obesity and periodontitis^46^. Moreover, peaks (52–54) displayed [M + H]^+^ at *m/z* (287.055, C_15_H_10_O_6_^+^) with MS^2^ fragments at *m/z* 265 and 275,( 303.05, C_15_H_10_O_7_^+^ ) with MS^2^ fragments at *m/z* 273 and 289 and (291.0863, C_15_H_14_O_6_^+^) with MS^2^ fragments at *m/z* 262 were assigned as 5, 7, 20, 30—tetrahydroxyflavone, 3,5,7,20,50—pentahydroxyflavone and ent-fisetinidol-4-β-ol, respectively according to^32^. Some of the flavonoidal compounds present in the leaves (compounds 22–28) in the negative ionization mode were found to be linked to one or more sugar residues such as galloyl derivatives of glucose in peaks 22 and 23 [M-H]^−^ which were annotated as galloyl hexoside (*m/z* 331.0682, C_13_H_16_O_10_^−^) with MS^2^ fragments at *m/z* 331,169,151, 125^42^ and ethyl-O-β-D-(6’-*O*-galloyl)- glucopyranoside (*m/z* 359.0999, C_15_H_20_O_10_^−^) with MS^2^ fragments at *m/z* 169.0140^43^, respectively. Additionally, five *O*-type flavonoid glycosides (compounds 24–28) were identified comprising apigenin-7-*O*-β-D-glucoside (*m/z* 431.0967, C_21_H_20_O_10_^−^) and along with fragment peak at 269.0439 based on result analysis from^35^. Additionally, the other *O*-type flavonoid glycosides were detected according to^47^ assigned as kaempferol-3-*O*-deoxyhexoside (*m/z* 431.0967, C_21_H_20_O_10_^−^) with MS^2^ fragments at *m/z* 431.1002, 285.0408, 284.0332, 255.0303, 227.0352 and 229.0506, kaempferol-3-*O*-hexoside (*m/z* 447.0917, C_21_H_20_O_11_^−^) with MS^2^ fragments at *m/z* 447.0945, 285.0398, 284.0318, 255.0292, 227.0340 and 151.0031, quercetin-3-*O*-hexoside (*m/z* 463.0868, C_21_H_20_O_12_^−^) with MS^2^ fragments at *m/z* 463.0899, 301.0355, 300.0271, 271.0247, 255.0295, 243.0296, 178.9985 and 151.0035 and myricetin-3-*O*-hexoside (*m/z* 479.0808, C_21_H_20_O_13_^−^) with MS^2^ fragments at *m/z*. 479.0841, 317.0286, 316.0221, 287.0193 and 271.0239 respectively. Quercetin-3-*O*-hexoside has been detected in several previous studies and has been reported as a potent antioxidant flavonidal compound^48^. In this study it was identified and detected for the first time in *M. caffra* leaf according to^47^.

#### Nitrogenous compounds

Nitrogen-containing metabolites were detected though at lower levels, among which l-tryptophan and gentiatibetine were characterized in this study (peak 56), (peak 55) respectively. [M_+_H]^+^ at *m/z* (205.0971, C_11_H_12_N_2_O_2_^+^) with MS^2^ fragments at *m/z* 188.0708, 146.0603 and 118.0654 was annotated as L-tryptophan based on result analysis from^49^. While gentiatibetine with [M_+_H]^+^ at *m/z* (166.0861, C_9_H_11_NO_2_^+^) exhibiting MS^2^ fragments at *m/z* 143,151 according to^32^.

#### Sphingolipids

Sphingolipids are a class of lipids with high structural diversity and biological pleiotropy. Three sphingolipid components were determined including dehydrophytosphingosine, octadecasphinganine and phytosphingosine by LC-MS/MS method Peaks 57, 58, and 59 exhibited molecular ions [M + H]^+^ at *m/z* 316.2843, 302.305 and 318.3 respectively, Most of the sphingolipids and their dihydro equivalents fragment to backbone ions with *m/z* 264 in positive ion mode as a key for the identification of sphingolipids in *Manilkara zapota* fruit^50^. Most notably, fragment ions (*m/z* 281, 280) are for dehydrophytosphingosine, whereas fragment ions at *m/z* 282, 264 correspond to phytosphingosine, moreover, fragment ions at *m/z* 285,284 are for octadecasphinganine. These metabolites are reported here for the first time in *caffra* leaf, and likely to account for a wide array of therapeutic indications such as treatment of cancer, inflammations, and metabolic disorders^51^.

#### In vitro antioxidant activity assays via DPPH free radical scavenging activity

**Fig. 5:**
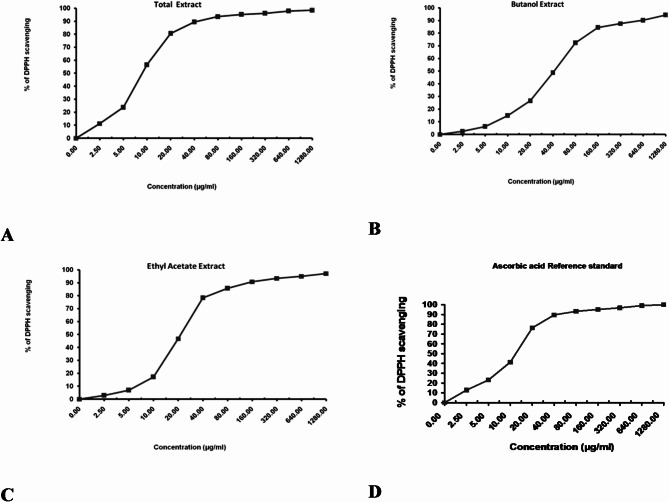
The radical DPPH scavenging activity, represented by percentage of inhibition, of the three different solvent extract compared to ascorbic acid. (**A**) Percentage of inhibition of total extract; (**B**) percentage of inhibition of *n*-butanol extract; (**C**) percentage of inhibition of ethyl acetate extract and finally; (**D**) percentage of inhibition of ascorbic acid.

The DPPH assay is a rapid and efficient method for evaluating free radical scavenging activity^29^, as it measures the ability of an extract to donate an electron or hydrogen radical to stabilize free radicals. In the present study, the antioxidant activity of *M. caffra* leaf extracts was assessed by measuring their percentage inhibition of DPPH radicals, as listed in Table S1. A total of eleven varying concentrations (0, 2.5, 5, 10, 20, 40, 80, 160, 320, 640, and 1280 µg/ml) of different solvent extract of *M. caffra* demonstrated different percentage of inhibition. The results demonstrated a concentration-dependent increase in scavenging activity across all extracts, with the highest inhibition observed at 1280 µg/ml. Among them, the crude methanol extract showed the highest scavenging activity (98.52%), followed by ethyl acetate (97.13%) and *n*-butanol fraction (94.38%). The antioxidant activity measurement has revealed that the crude methanol extract of *M. caffra* demonstrated greater antioxidant activity than ascorbic acid (Fig. 5A–D). previous reports have shown that methanol extract of medicinal plants possessed good pharmaceutical activity. Recent finding has reported that methanol extract of *M. caffra* leaves potentially have a free radical scavenging activity. Ethyl acetate extract has been used for extracting some phenolic and nitrogenous compounds. These compounds are known to scavange the free radicals and reactive oxygen species (ROS) including superoxide anion, hydroxyl radicals and singlet oxygen. Ethyl acetate extract of *M. caffra* showed a significant dose-dependent inhibition of DPPH activity. Among the two previous solvent extracts, crude methanol extract of *M. caffra* leaves exhibited highest potential antioxidant in a concentration dependent manner.

The IC_50_ value was calculated to determine the concentration of the sample required to inhibit 50% of free radical. The lower the IC_50_ value, the higher the antioxidant activity of samples. The observed IC_50_ value confirm that the crude methanol extract exhibited highest antioxidant activity followed by ethyl acetate extract and *n*-butanol extract, respectively (Table 4). Interestingly, the IC_50_ value of the crude methanol extract was also lower than ascorbic acid. According to^52^, extracts which possess IC_50_ values ranging from 50 to 100 mg/mL is considered to exhibit intermediate antioxidant activity. Meanwhile, extracts with IC_50_ value ranging between 10 and 50 mg/mL is considered to possess strong antioxidant activity (Table S3). In this case, the crude methanol extract, ethyl acetate and *n*-hexane extraxts possessed strong antioxidant activity.

**Table 4:** IC_50_ value of DPPH radical scavenging activity.

Sample	IC_50_ (µg/ml)
Total methanol extract	9± 0.37
Ethyl acetate extract	22.1 ± 0.79
*n*-Butanol extract	42.2 ± 1.65
Ascorbic acid	12.5 ± 0.7

Compared to other previously reported studies, *Mimusops elengi* Linn. (one of the most important species belonging to the Sapotaceae family) was reported for its strong antoxidant activity due to its high phenolic content^53^. The methanol extract of *M. elengi* bark showed marked inhibition 90.61% at a dose of 20 µg/mL and ascorbic acid as a reference compound showed marked inhibition 93.15%. Additionally, the DPPH assay was conducted with *M. caffra*,* M. Zeyheri*,* M. kummel*, and *M. laurifolia* at a concentration of 20 µg/ml^5^, revealing that the hydro-methanolic extracts exhibited scavenging effect of 67%, 56%, 42%, and 31%, respectively. The calculated IC_50_ value revealed that *M. caffra* was the best antioxidant among the previous species. A recent finding shows a marked inhibition 80.61% for the methanol extract of *M. caffra* leaf and 76.31% for standard ascorbic acid at the same dose. Therefore, the methanol extract of *M. caffra* leaf showed strong antioxidant activity by inhibiting DPPH radical scavenging activities when compared with standard ascorbic acid. Such activity of *M. caffra* owing to its richness in phenolic and flavonoid compounds which play a pivotal role in the antioxidant capacity. Although the antioxidant activities found in vitro experiment were only indicative of the potential health benefit, these results remain important as the first step in screening antioxidant activity of *M. caffra* leaf. Thus, it can be concluded that methanol extract of *M. caffra* leaf can be used as an accessible source of natural antioxidants with consequent health benefits.

#### In vitro anti-inflammatory activity via NO inhibitory effect activity

Nitric oxide (NO) is a pro-inflammatory mediator that plays a key role in the pathogenesis of inflammatory disorders, particularly when produced in excess underabnormal situations^54^. NO is synthesized and released into the endothelial cells by the help of nitric oxide synthases (NOSs), which convert arginine into citrulline generating NO in the process. NO has been recognized for its role invasodilatation in cardiovascular system. Additionally, it participates in immune responses through cytokine-activated macrophages, which release NO in high concentrations^55^.

Abnormal NO production is often associated with various animal and human diseases. In some cases, preventing a decrease in constitutive NO production in the vasculature may mitigate the development of vascular disease, while inhibition of uncontrolled NO production could also serve as a therapeutic target^56^. Although NO and other free radicals are generated in our body during inflammation for specific metabolic purposes, they also involved in regulation of cell growth, energy production and intercellular signaling. Thus, when an imbalance between free radical generation and body defence mechanisms occurs, free radicals can attack proteins in tissues, lipids in cell membranes, DNA and enzymes inducing oxidations, which cause protein modifications, membrane damage and DNA damage leading to inflammation and other series of human illnesses such as heart diseases and cancer^57^. Therefore, NO inhibitors represent important therapeutic advance in the management of inflammatory diseases, flavonoids were previously reported to have a markedly decline in NO production at higher doses as compared to control^58^. In the present study, there are many examples of flavonoids detected from the crude methanol extract of *M. caffra* leaf with anti-inflammatory activity (Tables 2, 3), such as quercetin^59^. Additionally, polyphenolic, proanthocyanidin, alkaloid, terpenoid and steroid compounds are usually responsible for the anti-inflammatory activities of plant extracts. These secondary metabolites act on different targets involved in the inflammatory pathway^57^. Among the phenolic content that have been detected in *M. caffra* leaf, gallic acid has received increasing attention for its powerful anti-inflammatory properties^60^.

**Fig. 6:**
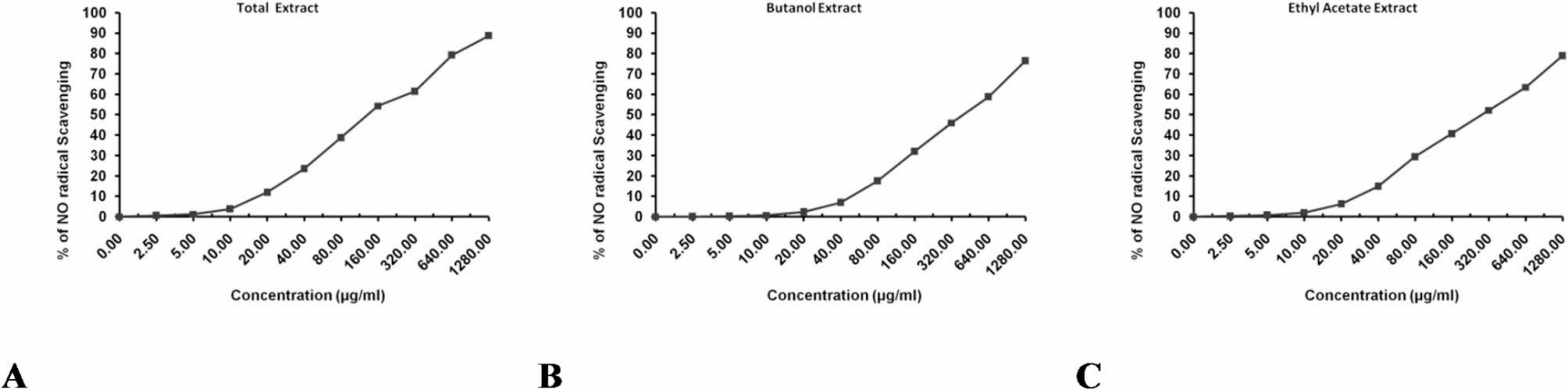
The radical NO scavenging activity, represented by percentage of inhibition, of the three different solvent extract compared to ascorbic acid. (**A**) Percentage of inhibition of total extract; (**B**) percentage of inhibition of *n*-butanol; and (**C**) percentage of inhibition of ethyl acetate extract.

In our study nitric oxide scavenging activity was evaluated using crude methanol extract, ethyl acetate and *n*-butanol fractions. The reductive potential of all three extracts exhibited dose dependent activity, as shown in Fig. 6. The IC_50_ values were calculated for all three extracts, Fig. 6A–C corresponding to 137.9 ± 2.76, 419.3 ± 7.89 and 289.6 ± 4.08 µg/ml. respectively. The results are summerized in Table S2 and graphically represented in Fig. 6. The regression analysis revealed a linear increase in % scavenging activity with increasing extract concentration for all three extracts (Fig. 6). These findings confirm that the the crude methanol extract showed potent anti-inflammatory activity compared to the ethyl acetate and *n*-butanol fractions. All three extracts showed good anti-inflammatory activity in relation to their total flavonoid and phenolic content.

## Conclusion

This study presents a comprehensive phytochemical profiling of *M. caffra* leaf extracts, targeting both volatile and non-volatile secondary metabolites using GC-MS and UPLC-MS/MS analyses. In vitro antioxidant and anti-inflammatory activity were assessed for the crude methanol extract, ethyl acetate and *n*-butanol fractions of *M. caffra* leaves by DPPH free radical scavenging and nitric oxide inhibition assays, respectively. A total of 50 volatile metabolites and 62 secondary metabolites were identified via GC-MS and UPLC-MS/MS, respectively, revealing a diverse range of bioactive constituents in the leaf extract. Notably, Phenolic and flavonoid compounds constituted about 32 compounds form the identified secondary metabolites by using UPLC-MS/MS analysis highlighting their abundance in *M. caffra* leaves. The DPPH radical scavenging assay and NO inhibition assay revealed that the crude methanol extract exhibited potential antioxidant with IC_50_ of 9 µg/ml and anti-inflammatory activity with IC_50_ of 137 µg/ml. These bioactivities are attributed to the richness with phenolic and flavonoid compounds detected via UPLC-MS/MS. The results of this study indicates that *M. caffra* crude methanol extract holds significant potential as a therapeutic agent for preventing or slowing aging and oxidative stress-related degenerative diseases. Additionally, ethyl acetate and *n*-butanol fractions are also exhibited good antioxidant activity, further supporting *M. caffra* as a valuable natural source of antioxidant. Further isolation and purification of specific bioactive metabolites from *M. caffra* leaves is recommended for future studies along with more indepth biological investigation. Moreover, green extraction techniques is recommended to be used instead of conventional solvent extraction such as electrochemical extraction, ultrasonic-assisted extraction, deep eutectic solvents, ionic liquids, enzyme-assisted extraction, microwave-assisted extraction and subcritical water extraction. In summary, *M. caffra* leaves represents a promising natural source of antioxidants and anti-inflammatory agents, with potential for development into the health-promoting dietary supplements.

## Materials and methods

### Plant material

*Mimusops caffra* E. Mey. ex A.DC leaf was collected from a tree growing in the Agricultural Research Center garden in Giza governorate (30.0209° N, 31.2113° E), Egypt, during in December 2020. The plant was botanically identified by Prof. Dr. Reem Samir Hamdy, Professor of Plant Taxonomy, Botany Department, Faculty of Science, Cairo University. Avoucher specimen was deposited in Pharmacognosy Department Herbarium, Faculty of Pharmacy, Egyptian Russian University under code (MCL1/21). About 10 Kg of *M. caffra* fresh leaves were washed with tap water and dried under shade.

### Plant extraction

The air-dried ground *M. caffra* leaves (100 g) were extracted with *n*-hexane as a nonpolar solvent which used later for study of volatile oil profile using GC-MS method. About 1400 g of *M. caffra* powder was subjected to cold maceration process in a conical flask using 100% methanol for 3 days at a room temperature. The extract was filtered using double ring 18.0 cm filter paper and the extraction process was repeated three times. The combined extracts were concentrated under reduced pressure at 55 °C by rotary evaporator in order to obtain the crude methanol extract (135 g). About 115 g of the crude extract were fractionated in separating funnel using solvents with different polarity as ethyl acetate, *n*-butanol, petrolium ether, and chloroform and each extract was prepared into final concentrations to yield 7 g, 18.5 g, 5 g and 7.5 g, respectively. The crude methanol extract, ethyl acetate, and *n*-butanol fractions were used subsequently in the investigation of antioxidant and anti-inflammatory activities.

### GC-MS analysis of volatiles in *M. caffra* leaf

Gas chromatography analysis was performed at Pharmacognosy Department, Faculty of Pharmacy, Ain Shams University, Cairo, Egypt on April 2021. *M. caffra n*-hexane extract was subjected to GC-MS analysis. *n*-Hexane is highly non-polar and volatile making it suited for extraction of non-polar volatile compounds while minimizing matrix interference in GC-MS analysis^61^. Mass spectra were recorded using Shimadzu GCMS-QP2010 (Koyoto, Japan) equipped with an Rtx-5MS fused bonded column (30 m × 0.25 mm i.d. X 0.25 μm film thickness) (Restek, USA) equipped with a split − splitless injector (1.0 µL of the prepared *n*-hexane extract was injected). The initial column temperature was kept at 50 °C for 3 min (isothermal) and programmed to 300 °C at a rate of 5 °C/min and kept constant at 300 °C for 10 min (isothermal). The injector temperature was 280 °C. The helium carrier gas flow rate was 1.37 mL/min. All the mass spectra were recorded by applying the following condition: (equipment current) filament emission current, 60 mA; ionization voltage, 70 eV; and ion source, 220 °C. Diluted samples (1% v/v) were injected with the split mode (split ratio 1: 15). Identification of volatile metabolites composition was performed by comparing their retention indices in relation to *n*-alkanes (C6 − C20), mass matching to NIST17, Wiley library database. Peaks were first deconvoluted using AMDIS software^10^.

### High-resolution ultra high-performance liquid chromatography analysis (UPLC-MS/MS)

About 100 mg of the crude methanol extract was dissolved in 5 mL 100% methanol and 3 µl was subjected to chromatographic separation using an I-Class UPLC system. The UHPLC analysis was performed on an Acquity UHPLC System (Waters) equipped with a HSS T3 column (100 × 1.0 mm, particle size 1.8 mm; Waters). The analysis was carried out by applying the following binary gradient at a flow rate of 150 mL min^− 1^: 0–1 min, isocratic 95% A (water/formic acid, 99.9/0.1 [v/v]), 5% B (acetonitrile/formic acid, 99.9/0.1 [v/v]); 1–16 min, linear from 5 to 95% B; 16–18 min, isocratic 95% B; and 18–20 min, isocratic 5% B. The injection volume was 3.1 mL (full loop injection). Eluted compounds were detected from m/z 90 to 1000 using a MicroTOF-Q hybrid quadrupole time-of flight mass spectrometer (Bruker Daltonics) equipped with an ApolloII electrospray ion source in negative and positive (deviating values in brackets) ion modes using the following instrument settings: nebulizer gas, nitrogen, 1.4 (1.6 bar); dry gas, nitrogen, 6.l min^− 1^, 190 °C; capillary, -5000 V (+ 4000 V); end plate offset, 500 V; funnel 1 RF, 200 Vpp; funnel 2 RF, 200 Vpp; in-source CID energy, 0 V; hexapole RF, 100 Vpp; quadrupole ion energy, 5 eV (3 eV); collision gas, argon; collision energy, 7 eV (3 eV); collision RF, stepping 150/350 Vpp (200/300 Vpp), (timing 50/50); transfer time, 58.3 µs; prepulse storage, 5 µs; pulser frequency, 10 kHz; and spectra rate, 3 Hz. Internal mass calibration of each analysis was performed by infusion of 20 µL 10 mM lithium formate in isopropanol : water, 1:1 (v/v), at a gradient time of 18 min using a diverter valve. For auto-MS/MS analysis, precursor ions were selected in Q1 with an isolation width of ± 3–10 Da and fragmented at collision energies of 15–70 eV using argon as a collision gas. Product ions detection was performed using the same settings as above, but with funnel 2 RF 300 Vpp in negative mode. Metabolites were characterized by their UV-vis spectra (210–650 nm), retention times relative to external standards, accurate MS and the domino MS/MS spectra in comparison to our in-house database, phytochemical dictionary of natural products database and reference literature.

### Antioxidant activity via DPPH radical scavenging activity

The antioxidant activity of extract was performed at the Regional Center for Mycology and Biotechnology (RCMB) at Al- Azhar University by using the DPPH free radical scavenging assay in triplicate and average values were considered^29^. Freshly prepared (0.004%w/v) methanol solution of 2,2-diphenyl-1-picrylhydrazyl (DPPH) radical was prepared and stored at 10 °C in the dark. A methanol solution of the test sample was prepared. A 40 uL aliquot of the methanol solution was added to 3 ml of DPPH solution. Absorbance measurements were recorded immediately with a UV-visible spectrophotometer (Milton Roy, Spectronic 1201). The decrease in absorbance at 515 nm was determined continuously, with data being recorded at 1 min intervals until the absorbance stabilized (16 min). The absorbance of the DPPH radical without antioxidant (control) and the reference compound ascorbic acid were also measured. All the determinations were performed in three replicates and averaged. The percentage inhibition (PI) of the DPPH radical was calculated according to the formula:

1 $${\text{PI }}={\text{ }}\left[ {\left\{ {\left( {{\text{AC}} - {\text{ AT}}} \right)/{\text{ AC}}} \right\}{\text{ }} \times {\text{ 1}}00} \right]$$

Where AC = Absorbance of the control at t = 0 min and AT = absorbance of the sample + DPPH at t = 16 min^62^.

The 50% inhibitory concentration (IC_50_), the concentration required to inhibit DPPH radical by 50%, was estimated from graphic plots of the dose response curve.

### Anti-inflammatory activity via nitric oxide (NO) inhibition activity

NO radical inhibition activity of the tested samples was determined according to method of Marcocci et al.^63^ by using a sodium nitroprusside (SNP). NO radical generated from SNP in aqueous solution at physiological pH reacts with oxygen to produce nitrite ions that were measured by the Greiss reagent. The reaction mixture (2 mL) containing various concentrations of the tested samples and SNP (10 mM) in phosphate buffered saline (PBS; pH 7.4) was incubated at 25 °C for 150 min. At the end of the incubation period, 1 mL of reaction mixture samples was diluted with 1 mL Greiss reagent (1% sulphanilamide (w/v) in 5% phosphoric acid (v/v) and 0.1% naphthyl ethylene diamine dihydrochloride). The mixture was incubated at 25 °C for further 30 min. The absorbance of these solutions was measured at 546 nm against the corresponding blank solution (without sodium nitroprusside). All the tests were performed in triplicate. The percent inhibition activity was calculated using the formula :

2 $$\:\text{I}\text{n}\text{h}\text{i}\text{b}\text{i}\text{t}\text{i}\text{o}\text{n}\:\text{\%}\:=\left[\frac{\text{A}\:\text{c}\text{o}\text{n}\text{t}\text{r}\text{o}\text{l}-\text{A}\:\text{s}\text{a}\text{m}\text{p}\text{l}\text{e}}{\text{A}\:\text{c}\text{o}\text{n}\text{t}\text{r}\text{o}\text{l}}\right]\times\:100$$

where, A control is the absorbance of the control reaction at 546 nm and Atest represents the absorbance of a test reaction at the same wavelength. Tested material concentration providing 50% inhibition (IC_50_) was calculated from the graph plotting inhibition percentage against concentration^64^.

### Statistical analysis

The results of biological investigation were analyzed in triplicate and displayed as average ± standard deviation of the mean (SD) (Tables S1 and S2). By using the t-tests analysis comparing each extract to ascorbic acid in DPPH assay significant differences (*p* < 0.05) appear at lower concentrations (≤ 80 µg/ml), where ascorbic acid retains higher activity. In NO inhibition assay, significant differences (*p* < 0.05) were observed at all concentrations, and crude methanol extract had significantly higher NO inhibition.

## Electronic supplementary material

Below is the link to the electronic supplementary material.

Supplementary Material 1

## Data Availability

All data generated or analyzed during this study are included in this published article [and its supplementary information files].
