# Supplementary material for: Metabolites profiling of Mimusops caffra leaf via multiplex GC-MS and UPLC-MS/MS approaches in relation to its antioxidant and anti-inflammatory activities
Source: Sci Rep. 2025 Apr 29;15:15072. doi: 10.1038/s41598-025-97161-6 (PMC12041594; doi:10.1038/s41598-025-97161-6)
Supplement: Supplementary file 1 — Supplementary Material 1 [file 41598_2025_97161_MOESM1_ESM.docx]

**Supplementary data**

**Metabolites profiling of *Mimusops caffra* leaf *via* multiplex GC-MS and UPLC-MS/MS approaches in relation to its antioxidant and anti-inflammatory activities**

**Mostafa H. Baky^1*^, Sara M. Rashad^1^, Omayma D. Elgindi^1^, Safwat A. Ahmed^2^**

*^1^ Department of Pharmacognosy, Faculty of Pharmacy, Egyptian Russian University, Badr City, Cairo, Egypt.*

*^2^Department of Pharmacognosy, Faculty of Pharmacy, Suez Canal University*

*Corresponding author:

**Mostafa H. Baky**

*Pharmacognosy Department, College of Pharmacy, Egyptian Russian University, Badr City 11829, Cairo, Egypt.*
E-mail addresses: [dr_mostafa1984@yahoo.com](mailto:dr_mostafa1984@yahoo.com), [mostafa-hasan@eru.edu.eg](mailto:mostafa-hasan@eru.edu.eg)

Tel: +201007906443

| **Table** S1. Percentage of DPPH scavenging inhibition in *Mimusops caffra* extracts | | | | | | | | |
| --- | --- | --- | --- | --- | --- | --- | --- | --- |
|  | **Crude methanol extract** | | ***n*-Butanol extract** | | **Ethyl acetate fraction** | | **Ascorbic acid Reference standard** | |
| **Sample conc. (µg/ml)** | **DPPH scavenging %** | **S.D. (±)** | **DPPH scavenging %** | **S.D. (±)** | **DPPH scavenging %** | **S.D. (±)** | **DPPH scavenging %** | **S.D. (±)** |
| 1280 | 98.52 | 0.34 | 94.38 | 0.46 | 97.13 | 0.75 | 99.87 | 0.31 |
| 640 | 97.89 | 0.57 | 90.24 | 0.28 | 95.02 | 0.64 | 98.96 | 0.68 |
| 320 | 96.14 | 0.32 | 87.61 | 0.53 | 93.46 | 0.92 | 96.72 | 1.44 |
| 160 | 95.26 | 0.65 | 84.56 | 0.68 | 90.82 | 0.76 | 95.04 | 0.98 |
| 80* | 93.58 | 0.34 | 72.31 | 1.47 | 85.74 | 1. 32 | 93.17 | 1.75 |
| 40* | 89.47 | 0.45 | 48.69 | 2.75 | 78.36 | 2.93 | 89.48 | 2.36 |
| 20* | 80.61 | 0.37 | 26.58 | 1.78 | 46.71 | 3.45 | 76.31 | 3.89 |
| 10* | 56.53 | 2.91 | 14.86 | 0.94 | 17.15 | 1.37 | 41.36 | 4.28 |
| 5* | 23.74 | 3.22 | 6.24 | 0.62 | 6.93 | 0.61 | 23.15 | 2.37 |
| 2.5* | 11.28 | 1.64 | 2.43 | 0.21 | 2.87 | 0.45 | 12.84 | 1.42 |
| 0 | 0 |  | 0 |  | 0 |  | 0 |  |

* significant differences (p < 0.05) appear at lower concentrations (≤ 80 µg/ml).

| **Table S2**. Percentage of NO radical inhibition in *Mimusops caffra* extracts | | | | | | |
| --- | --- | --- | --- | --- | --- | --- |
|  | **Crude methanol extract** | | ***n*-Butanol fraction** | | **Ethyl acetate fraction** | |
| **Sample conc. (µg/ml)** | **DPPH scavenging %** | **S.D. (±)** | **DPPH scavenging %** | **S.D. (±)** | **DPPH scavenging %** | **S.D. (±)** |
| 1280* | 88.74 | 1.42 | 76.46 | 2.68 | 78.95 | 1.43 |
| 640* | 79.23 | 2.05 | 58.82 | 3.46 | 63.46 | 1.72 |
| 320* | 61.48 | 1.68 | 46.03 | 1.95 | 52.19 | 2.53 |
| 160* | 54.29 | 2.67 | 31.94 | 2.32 | 40.67 | 1.49 |
| 80* | 38.76 | 1.43 | 17.48 | 1.74 | 29.41 | 0.97 |
| 40* | 23.51 | 0.79 | 6.95 | 0.86 | 14.85 | 0.32 |
| 20* | 11.94 | 0.38 | 2.34 | 0.62 | 6.23 | 0.49 |
| 10* | 3.75 | 0.61 | 0.69 | 0.17 | 1.89 | 0.15 |
| 5* | 1.23 | 0.09 | 0.28 | 0.04 | 0.74 | 0.28 |
| 2.5* | 0.62 | 0.14 | 0.07 | 0.05 | 0.31 | 0.17 |
| 0 | 0 |  | 0 |  | 0 |  |

* Significant differences (p < 0.05) were observed at all concentrations.

| **Table S3**. Antioxidant Activity according to ([Martiningsih, Mudianta et al. 2021](file:///C:\Users\user\Desktop\2025\resala\Revised%20M.%20Caffra%20manuscript%20MB%207-1-2025.docx#_ENREF_31)) | |
| --- | --- |
| **IC50 (µg/ml)** | **Mark** |
| < 10 μg/mL | Very strong Antioxidant Activity |
| 10-50 μg/mL | Strong Antioxidant Activity |
| 50-100 μg/mL | Moderate Antioxidant Activity |
| 100-250 μg/mL | Weak Antioxidant Activity |
| > 250 μg/mL | Inactive Antioxidant Activity |
